# Are They Calling My Name? Attention Capture Is Reflected in the Neural Tracking of Attended and Ignored Speech

**DOI:** 10.3389/fnins.2021.643705

**Published:** 2021-03-22

**Authors:** Björn Holtze, Manuela Jaeger, Stefan Debener, Kamil Adiloğlu, Bojana Mirkovic

**Affiliations:** ^1^Neuropsychology Lab, Department of Psychology, University of Oldenburg, Oldenburg, Germany; ^2^Fraunhofer Institute for Digital Media Technology IDMT, Division Hearing, Speech and Audio Technology, Oldenburg, Germany; ^3^Research Center for Neurosensory Science, University of Oldenburg, Oldenburg, Germany; ^4^Cluster of Excellence Hearing4all, University of Oldenburg, Oldenburg, Germany; ^5^HörTech gGmbH, Oldenburg, Germany

**Keywords:** EEG, speech envelope tracking, auditory attention decoding (AAD), ignored speech processing, attention capture, own name, P3, steering beamformer

## Abstract

Difficulties in selectively attending to one among several speakers have mainly been associated with the distraction caused by ignored speech. Thus, in the current study, we investigated the neural processing of ignored speech in a two-competing-speaker paradigm. For this, we recorded the participant’s brain activity using electroencephalography (EEG) to track the neural representation of the attended and ignored speech envelope. To provoke distraction, we occasionally embedded the participant’s first name in the ignored speech stream. Retrospective reports as well as the presence of a P3 component in response to the name indicate that participants noticed the occurrence of their name. As predicted, the neural representation of the ignored speech envelope increased after the name was presented therein, suggesting that the name had attracted the participant’s attention. Interestingly, in contrast to our hypothesis, the neural tracking of the attended speech envelope also increased after the name occurrence. On this account, we conclude that the name might not have primarily distracted the participants, at most for a brief duration, but that it alerted them to focus to their actual task. These observations remained robust even when the sound intensity of the ignored speech stream, and thus the sound intensity of the name, was attenuated.

## Introduction

When listening to continuous speech, the listener’s brain activity synchronizes to the slow amplitude fluctuation of that speech signal, i.e., the speech envelope (Aiken and Picton, 2008). Interestingly, when selectively attending to one among other speech streams, the listener’s brain activity synchronizes most effectively to the envelope of the attended speech stream (Ding and Simon, 2012; Mesgarani and Chang, 2012). This synchronization has often been exploited to investigate selective auditory attention. It has, however, consistently been observed that not only the attended but also the ignored speech envelope correlates with the listener’s brain activity, albeit to a smaller extent (Kong et al., 2014; Olguin et al., 2018). Moreover, challenges in auditory selective attention have been linked to deficits in suppressing the ignored auditory background (Petersen et al., 2017), clearly indicating the necessity to investigate the neural representation of ignored speech.

Over the past few years, different linear and non-linear methods have been proposed to relate the speech envelope to the listener’s concurrent brain activity (for a review of linear methods, see Alickovic et al., 2019; and for non-linear methods, de Taillez et al., 2017 and Ciccarelli et al., 2019). These methods are commonly referred to as auditory attention decoding (Crosse et al., 2016; O’Sullivan et al., 2017) or speech envelope tracking (Giraud and Poeppel, 2012). Initially, research on the neural tracking of speech has focused on identifying the attended among all present speech streams, by finding the speech stream whose feature correlates the most to the neural signal (Mirkovic et al., 2015; O’Sullivan et al., 2015). These findings are crucial for the development of neuro-steered hearing aids (Aroudi et al., 2019; Geirnaert et al., 2020). In parallel, some other research has been conducted in which the neural tracking of speech was used to investigate the underlying principles of speech perception, such as the encoding of phonemes (Di Liberto et al., 2018) or semantic dissimilarity (Broderick et al., 2018).

However, these studies mainly concentrated on attended speech, and only recently, speech envelope tracking was adopted to investigate the processing of ignored speech. To give an example, it has been observed that the number of ignored speech streams (Hambrook and Tata, 2019) and its linguistic content (Olguin et al., 2018) affect the comprehension of attended speech. Therefore, it is important to explore the interference caused by ignored speech and, in particular, the processes which occur when ignored speech involuntarily captures one’s attention. In a recently published study, Hambrook and Tata (2019) observed a decrease in the neural tracking of an attended speaker whenever content from an ignored speaker intruded the listener’s perception. This result was found for simple, unconnected sentences of repetitive structure. Similar results were obtained in a study conducted by Huang and Elhilali (2020), in which participants were instructed to attend to an artificial tone sequence while ignoring a simultaneously presented auditory background stream which included salient events. In addition, Huang and Elhilali (2020) found an increase in the neural tracking of the auditory background after highly salient events were presented therein. Both studies suggest that ignored auditory streams can involuntarily capture a listener’s attention, which fits into the larger framework of bottom-up attention concepts (Bronkhorst, 2015; Kaya and Elhilali, 2017). Here, we investigate whether similar observations can be made in a competing-speaker paradigm, that is, in ecologically realistic scenarios of two (or more) continuous speech streams.

The main objective of the current study was to employ speech envelope tracking to shed more light on the attentional effect of relevant events embedded in a complex, to-be-ignored speech stream. We implemented a competing-speaker paradigm in which participants were simultaneously presented with two continuous narratives, each narrated by a different male speaker. The participants’ task was to attend to one of them and to ignore the other, while we measured brain activity using electroencephalography (EEG). Unknown to the participants, their first name was embedded into the to-be-ignored narrative as a potentially attention-capturing event (Moray, 1959; Wood and Cowan, 1995). Thus, instead of a physically salient event as used by Huang and Elhilali (2020), we used a semantically, personally relevant event. To estimate whether participants detected their name, we examined the event-related potential (ERP). It is known that hearing the own name elicits a P3 ERP component (Berlad and Pratt, 1995; Perrin et al., 2005), which we analyzed to determine whether the presentation of one’s name in the to-be-ignored speech stream transiently captured the listener’s attention. Given the capacity-limited nature of attentional processes (Lavie et al., 2014), we expected a transient decrease in the neural tracking of the to-be-attended speech stream as well as a transient increase in the neural tracking of the to-be-ignored speech stream if the name had attracted the participant’s attention toward the to-be-ignored stream. In addition, we were interested in whether this pattern would remain robust even if the speech intelligibility of the to-be-ignored speech stream, and thus, the detectability of one’s name, was significantly reduced. Therefore, in one condition, the to-be-attended and to-be-ignored speech streams were presented equally loud, whereas in the other condition, the to-be-attended speech stream was placed more strongly in the foreground by attenuating the sound intensity of the to-be-ignored speech stream. We expected participants to detect their name less often when the to-be-ignored speaker was attenuated.

## Materials and Methods

### Participants

Twenty-five native German speakers (mean age 25.24 ± 6.42 years, 15 females) without a psychological or neurological condition were participated in the current study. Two of these had to be excluded as they did not meet the requirements of normal hearing, i.e., a bilateral hearing threshold of 20 dB or better for octave frequencies from 0.125 to 8 kHz (World Health Organization, 2001). One additional participant had to be excluded due to an asymmetry in hearing thresholds of more than 5 dB between the left and the right ear for multiple octave frequencies. A fourth participant had to be excluded due to excessive movement during the EEG measurement, resulting in a total of 21 included participants (mean age 24.19 ± 3.93 years, 14 females). To decrease stimulus heterogeneity across participants, we exclusively recruited participants with a first name containing two or three syllables. The study was approved by the local ethics committee (University of Oldenburg, Germany, Drs.EK/2019/006). All participants signed a written informed consent before participating and received a monetary compensation of €8 per hour.

### Paradigm

Participants performed a competing-speaker paradigm in which they were presented with two concurrent narratives, each narrated in German by a different male speaker. Their task was to attend to one of them and to ignore the other, as indicated by the experimenter. Participants were instructed to attend to the same speaker over the entire experiment, while the to-be-attended speaker was pseudo-randomized across participants. Unknown to the participants, their first name was occasionally presented, embedded in the to-be-ignored narrative.

### Stimuli

#### Narratives

Two narratives were selected which had previously been used in Mirkovic et al. (2016) and Jaeger et al. (2020). Within these narratives, silent periods exceeding 0.5 s had been shortened to 0.5 s to reduce the chance of participants switching to the other narrative during a period of long silence. To ensure equal speech intelligibility of both speakers, the narratives’ root mean square (RMS) had been balanced as described in Mirkovic et al. (2016). For audio presentation, the narratives were resampled to 32 kHz and divided into five blocks of 10 min duration each.

#### Names

Audio files containing the participant’s first name were generated using the text-to-speech converter available on www.notevibes.com (Notevibes, 2019). We selected the German voice “Markus” as it best resembled the pitch of both narrative speakers. Silent periods before the name onset were removed so that the generated name audio file immediately started with the name onset. We increased the speaking rate of the name audio file by 15% to match it to the playback speed of both narratives. This change in playback speed was done without a change in the speaker’s pitch, using the audio processing software Audacity (Audacity 2.3.2; Audacity Team, Pittsburgh, PA, United States). Increasing the playback speed resulted in name audio files ranging from 379 to 597 ms, depending on the length of the name. These audio files were subsequently resampled to 32 kHz using MATLAB custom scripts (MATLAB R2018a; The MathWorks, Natick, MA, United States).

#### Name Embedment

**FIGURE 1:**
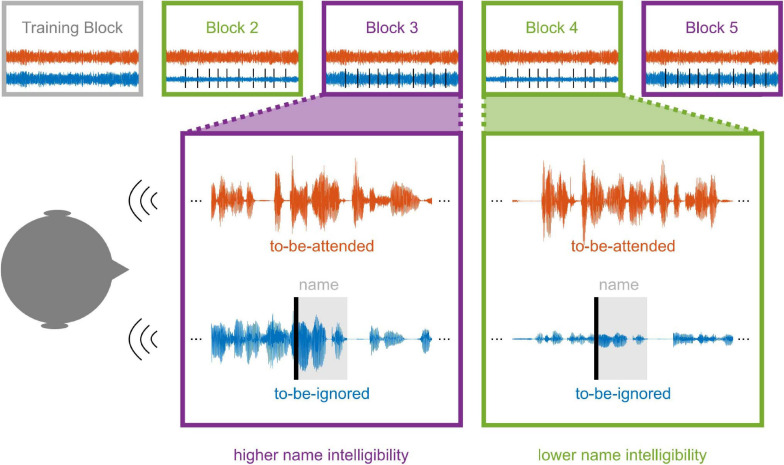
Illustration of the paradigm. The paradigm consisted of five 10-min blocks, in which participants were presented with two concurrent narratives. Participants were instructed to attend to the same narrative throughout the experiment. In all but the first 10-min block, the participant’s name was embedded 10 times within the to-be-ignored narrative (name onset indicated as vertical black bar). In the first 10-min block, both narratives were presented at equal sound intensity. Two of the remaining four blocks were presented in the “higher name intelligibility” condition, in which both narratives were presented at equal sound intensity. The other two blocks were presented in the “lower name intelligibility” condition, in which the to-be-ignored narrative, and thus the participant’s name, was attenuated. Conditions were always alternated for the last four blocks. One half of the participants started with the higher and the other half with the lower name intelligibility condition.

In each, but the first 10-min block, the participant’s name was presented 10 times, embedded within the to-be-ignored narrative (Figure 1). Thus, participants were presented 40 times with their name across the entire experiment. Time points at which the name occurred were identical for those participants attending to the same narrative. These time points were carefully chosen in advance and met the following criteria: a participant’s name did neither occur in the first minute nor in the last 30 s of a 10-min block and two name occurrences were at least 30 s apart. In addition, the participant’s name always replaced a word at the end of a sentence to maintain the narrative’s speech rhythm. This also allowed names of different lengths to be embedded due to the pause at the end of a sentence. However, applying these criteria did not always allow us to create semantically correct sentences. Therefore, we always replaced a verb at the end of the sentence to keep semantic violation constant over all name occurrences. With regard to the implementation, the respective narrative was faded out for 20 ms before the onset of the to-be-replaced verb, then muted for 700 ms, and faded back in for 20 ms. Before adding the participant’s name at the onset of the replaced verb, the RMS of the name audio file was adjusted to the to-be-ignored narrative’s RMS of the 2 s preceding the replaced verb to avoid sudden changes in sound intensity. The RMS of the two preceding seconds was only calculated after removing all its silent periods as the name audio file also did not include silent periods. As a control, for each of the last four 10-min blocks, we selected 10 nouns which occurred at the end of a sentence within the to-be-ignored speech stream. Again, a control word did neither occur in the first minute nor in the last 30 s of a 10-min block and two control words were at least 30 s apart. In addition, we ensured that a control word was at least 1.5 s away from a name occurrence.

#### Stimuli Presentation

The two concurrent narratives were presented to the participants via custom-made behind-the-ear hearing aid dummies (Ear-Technic, Istanbul, Turkey). The sound was produced by a receiver (Receiver E50DA028; Sonion, Roskilde, Denmark) within the hearing aid dummy and transmitted via a plastic tube to the participant’s ear canal. To attenuate noise from the environment, participants were seated in a soundproof cabin. The audio processing before presentation was done outside the hearing aid dummy on an Intel NUC computer with a Core i7 processor and an RME Fireface UCX sound card using the open-source, real-time capable audio signal processing platform open master hearing aid (openMHA; Herzke et al., 2017). To separate both narratives in virtual space, the audio signal of each narrative was convolved with a head-related impulse response function from Kayser et al. (2009), corresponding to −30° and 30°, respectively. As a result, participants perceived the one narrative to originate from their front left and the other narrative to originate from their front right.

#### Listening Conditions

To investigate whether one’s name induces an attention-capturing effect even if the to-be-ignored narrative is attenuated, participants performed the competing-speaker paradigm in two conditions. In one condition, the to-be-attended and to-be-ignored narratives were presented at equal sound intensity. In the other condition, the speech intelligibility of the to-be-attended narrative was enhanced by attenuating the sound intensity of the to-be-ignored narrative, including the participant’s name. As attenuating the to-be-ignored narrative reduced its speech intelligibility and thus the intelligibility of one’s name, the later condition was termed “lower name intelligibility,” whereas the former condition was termed “higher name intelligibility.” Note that differences in intelligibility were not explicitly tested but inferred from the different signal-to-noise ratios between the to-be-attended and to-be-ignored speech stream. Attenuating the to-be-ignored narrative was accomplished using the steering beamformer algorithm described in Adiloğlu et al. (2015). The attenuation was frequency dependent such that some frequencies of the narratives’ speakers were more strongly attenuated than others (Supplementary Figure 1). Thus, both speakers were attenuated differently by the same beamforming algorithm due to their different frequency composition and due to the asymmetric nature of the head-related impulse response functions. However, on a subjective level, both speakers were perceived as equally loud when attenuated. For all participants, in the first 10-min block, both narratives were played at equal sound intensity without the name being embedded in the to-be-ignored narrative. For the subsequent four 10-min blocks, the lower and higher name intelligibility conditions were alternated with the condition order pseudo-randomized over participants (Figure 1).

### Data Acquisition

#### Behavioral Data

To motivate participants and to behaviorally evaluate whether they paid attention to their assigned narrative, participants had to answer a content questionnaire after each 10-min block. This content questionnaire consisted of 10 multiple-choice questions, each approximately covering the content of a 1-min segment. Important to note, these questions did not necessarily refer to content presented immediately after the name and thus did not serve as behavioral measure of the name’s distraction effect. Each multiple-choice question included four possible options with only one of them being correct, plus a fifth option, stating “I don’t know.”

At the end of the experiment, participants were given an additional questionnaire to retrospectively assess the number of detected names. First, participants had to answer whether they noticed their name to be presented at all in the to-be-ignored narrative. If they answered with yes, they had to indicate on a scale from 1 to 10 how often per 10-min block they detected their name. Second, they were asked whether they noticed that in two out of the last four 10-min blocks, both narratives were presented at equal volume (higher name intelligibility), while in the remaining two 10-min blocks, the to-be-attended narrative was louder than the to-be-ignored narrative (lower name intelligibility). If they did, they were asked the following questions: (1) How often did you hear your name per 10-min block, only concerning the two blocks in which both narratives were presented at equal volume? (2) How often did you hear your name per 10-min block, only concerning the two blocks in which the narrative you attended to was louder than the one you ignored? To answer each question, respectively, a scale ranging from 0 to 10 was provided. Here, we included the value 0 as participants might have only heard their name in one but not in the other condition.

#### Neurophysiological Data

Neurophysiological data were recorded with an equidistant 64-channel Ag/AgCl EEG cap (Easycap GmbH, Hersching, Germany), including a fronto-polar ground electrode, a reference electrode positioned on the tip of the nose, and two electrooculogram (EOG) electrodes, one positioned below each eye. Impedances were kept below 20 kΩ using Abralyt HiCl gel (Easycap GmbH). We intentionally did not collect data from the five most occipital electrodes due to their large distance to the scalp as well as from 10 electrodes around the ears due to concurrent around-the-ear EEG recording with cEEGrids (Debener et al., 2015). Results obtained with cEEGrid will be presented elsewhere. EEG cap data were sampled at 500 Hz using a stationary BrainAmp amplifier (Brain Products GmbH, Gilching, Germany) with a recording band-pass filter from 0.0159 to 250 Hz. To reduce artifacts, participants were instructed to direct their gaze to a white fixation cross on a gray computer screen in front of them and to move as little as possible.

### Data Analysis

#### Behavioral Data

With respect to the content questionnaire, each question was either marked as correct or incorrect, considering “I don’t know” as incorrect. Subsequently, a percentage of correctly answered questions was calculated per participant. Regarding the retrospective estimate of detected names, for each participant, we obtained one value per condition. If participants did not notice that their name was presented at all, both values were set to zero. If participants did not notice any difference between the two conditions in terms of the sound intensity, both values were set to the initial value concerning all four blocks. If both previously mentioned facts were noticed, the value obtained per condition was directly used. The difference between these values was examined using a one-sided Wilcoxon signed-rank test as we expected more names to be detected in the higher than in the lower name intelligibility condition. The average of these two condition-independent values was used for all subsequent condition-independent analyses.

#### Neurophysiological Data

Before the EEG data analysis, we accounted for a constant delay of 102 ms between the EEG data and the event marker stream, which in turn contained the onsets of each 10-min block. All analysis steps were performed in EEGLAB v13.6.5b (Delorme and Makeig, 2004) and implemented in MATLAB R2019b (The MathWorks, Natick, MA, United States). MATLAB code used to compute the results presented in the current study can be found on GitHub^1^. For artifact correction, data were first low-pass filtered with a pass-band edge of 40 Hz and then high-pass filtered with a pass-band edge of 2 Hz (*pop_eegfiltnew*). Data were then epoched into consecutive 1-s segments. Segments containing atypical artifacts were rejected using the build-in EEGLAB function *pop_jointprob* (local and global threshold: 2 SDs). Subsequently, data were decomposed, running an independent component analysis (ICA), and components containing stereotypical artifacts (e.g., eye blinks, heartbeat, etc.) were identified by visual inspection. The computed ICA weights were then applied to the unfiltered raw data and all but the artifactual components were back-projected. On average, per participant 7.78% of all components were identified as artifactual, ranging from 2 out of 49 components in the best case to 6 out of 49 components in the worst case.

##### P3 Component

Regarding the P3 analysis, artifact-corrected data were first low-pass filtered with a pass-band edge of 10 Hz (Hamming windowed FIR filter of order 660) and then high-pass filtered with a pass-band edge of 0.1 Hz (Hamming windowed FIR filter of order 16,500). Thereafter, data were epoched from −500 to 1,500 ms relative to the name and control word onset and then baseline corrected from −500 to 0 ms. This resulted in 80 epochs per participant, 40 for the name occurrences and 40 for the control words, each set consisting of 20 epochs per condition. Due to the low number of epochs per participant and condition, each epoch contributed strongly to the participant’s ERP. Therefore, it was important to reject epochs containing artifacts which our artifact correction procedure was not able to account for. For this epoch rejection, we used the TBT plugin (version 2.5.0; Ben-Shachar, 2018) in EEGLAB with a min/max threshold of 150 μV. If two or less channels in an epoch exceeded this criterion, the respective channels were interpolated in that epoch, whereas the entire epoch was rejected if more than two channels exceeded this criterion. An entire channel was interpolated over all epochs if it exceeded the criterion in more than 30% of all epochs. As a result, over all participants, a single channel was interpolated in 15 epochs, 16 epochs were completely rejected, and no channel was interpolated over all epochs. For all condition-independent analyses, epochs for the lower and higher name intelligibility condition were pooled, whereas they were kept separate for the condition-dependent analyses. After generating the participants’ ERPs for the name and control word, respectively, we smoothed them with a moving average filter of 100 ms to get a more accurate estimate of the P3 latency. To statistically test for the presence of a condition-independent P3 component, we calculated the mean amplitude over the time window from 500 to 1,200 ms. This time window was estimated based on the morphology and topography of the grand average ERP in response to the name. The mean amplitude over this time window was then compared between the name and control word ERPs using a one-sided Wilcoxon signed-rank test. Thereafter, the individual condition-dependent and condition-independent P3 latencies in response to the name were determined as the latency of the ERP’s maximum peak in the time window from 500 to 1,200 ms after name onset. The individual P3 amplitudes were calculated as the mean amplitude at the individual P3 latency ±50 ms. To statistically compare the P3 amplitude and latency across conditions, a one-sided Wilcoxon signed-rank test was applied as we expected a higher P3 amplitude as well as a shorter P3 latency for the higher name intelligibility condition. In addition, we investigated the condition-independent relation between the subjectively reported number of detected names and the P3 amplitude in response to the name. Here, the underlying assumption was that in epochs where the name was not detected, no P3 component was elicited. Consequently, these epochs would decrease the P3 amplitude of a participant’s ERP averaged over all name occurrences. Thus, we expected a positive relation between the subjectively reported number of detected names and the P3 amplitude of the participant’s ERP, which we tested with a one-sided Spearman rank correlation.

##### Speech Envelope Tracking

To extract the speech envelopes of both narratives, we implemented the procedure described in Petersen et al. (2017). In short, we first calculated the absolute values of the narrative’s Hilbert transform, which we then low-pass filtered at 15 Hz. To accentuate word and syllable onsets, we took the first derivative of the low-pass filtered speech signal and half-wave rectified it. Lastly, we downsampled the speech envelope to 500 Hz to match it to the sampling rate of the EEG data.

For speech envelope tracking, the artifact-corrected EEG data were re-referenced to the common average, low-pass filtered with a pass-band edge of 15 Hz (Hamming windowed FIR filter of order 440), and then high-pass filtered with a pass-band edge of 1 Hz (Hamming windowed FIR filter of order 1,650). Conceptually, we followed the cross-correlation approach proposed by Horton et al. (2013), in which the filtered EEG signal of each channel was cross-correlated with the to-be-attended and to-be-ignored speech envelope, respectively. Then, to obtain a more robust measure of attention, we calculated the SD of cross-correlation functions over channels to estimate the cross-correlation magnitude as a function of time lag. In general, taking the SD over channels gives a root-mean-square or global field power (GFP) value which measures the magnitude of a signal across all channels, at each point in time (Murray et al., 2008). By applying this procedure, channel selection and multiple comparison problems were avoided. To validate our magnitude-oriented cross-correlation approach, we first compared its results with findings obtained in previous speech envelope tracking studies. To this end, the filtered EEG data of the last four 10-min blocks were segmented into consecutive 5-s segments which were then baseline corrected by subtracting the mean of the respective EEG data segment. It is important to note that these segments were not time-locked to name onsets. Here, 5-s segments were chosen as this time window has been shown to constitute the highest temporal resolution which still produces reliable results when using cross-correlation for speech envelope tracking (Jaeger et al., 2020). The speech envelopes of the to-be-attended and to-be-ignored narratives were also segmented into consecutive 5-s segments. Thereafter, each channel of a 5-s EEG data segment was cross-correlated with the corresponding 5-s segment of the to-be-attended and to-be-ignored speech envelope at different time lags ranging from −1,000 to 1,000 ms. The resulting cross-correlation functions were then averaged over all 5-s segments, resulting in two sets of cross-correlation functions per participant—one set for the to-be-attended speech envelope and another set for the to-be-ignored speech envelope, with each set containing cross-correlation functions of individual channels. To estimate the cross-correlation magnitude at different time lags, we then calculated the SD over channels for each set. This resulted in two cross-correlation magnitude functions per participant. As a control, a third cross-correlation magnitude function was calculated in which the 5-s segments of the to-be-attended speech envelope were cross-correlated with non-matching EEG data segments. Cross-correlation magnitude functions and the topographic organization of cross-correlation values at prominent time lags were inspected for attention effects observed in previous studies. To quantify the cross-correlation magnitude irrespective of specific time lags, we averaged the cross-correlation magnitude values at time lags from 0 to 500 ms. These obtained values were statistically compared between the to-be-attended, to-be-ignored, and control speech envelope using one-sided Wilcoxon signed-rank tests.

**FIGURE 2:**
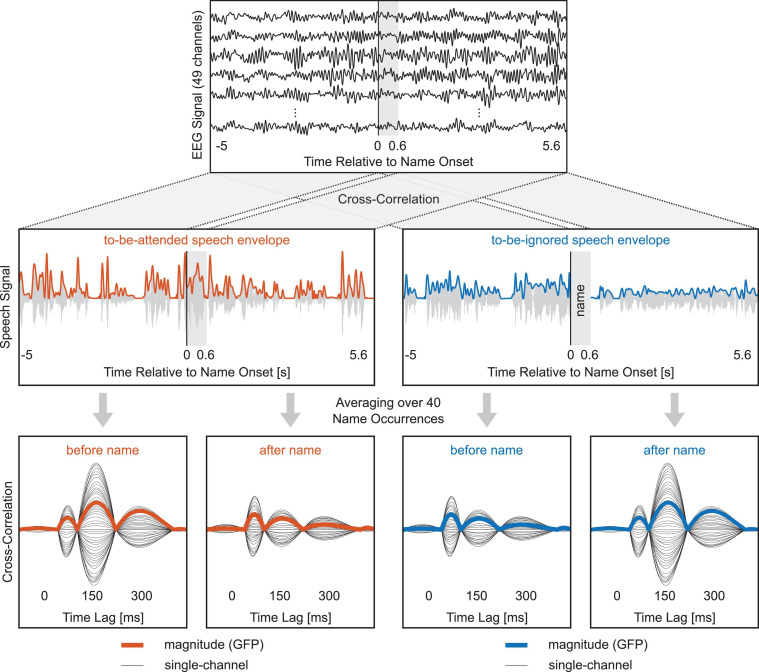
Speech envelope tracking of a single participant relative to name onset. Top row reflects single EEG channels of a segment before and after a name occurrence. Middle row shows the corresponding speech envelope segments of the to-be-attended and to-be-ignored narrative. Cross-correlating the speech envelope segments with all channels of the corresponding EEG segments resulted in four sets of cross-correlation functions for each name occurrence—two sets for the to-be-attended speech envelope (before and after) and two sets for the to-be-ignored speech envelope (before and after), with each set containing the cross-correlation function of all single EEG channels. Subsequently, these four sets were generated for and then averaged over all name occurrences (bottom row). Black lines in the bottom row reflect cross-correlation functions of single EEG channels averaged over all name occurrences for one participant. Colored lines indicate the cross-correlation magnitude functions of one participant, calculated as the SD over channels (GFP).

To evaluate the speech envelope tracking of both narratives relative to the name, a similar procedure as the one described above was performed. The only difference was that now the 5-s segments were selected relative to the name onset (Figure 2). In other words, the 5-s segment immediately before the name occurrence started at −5 s relative to the name onset and ended at the name onset. The 5-s segment immediately after the name occurrence started at 0.6 s and ended at 5.6 s relative to the name onset. The 0.6-s segment containing the participant’s name was cut out so that the speech envelope segments before and after the name occurrence were identical across participants. To visualize the temporal evolution of cross-correlation magnitude values relative to the name, we created six consecutive 5-s segments before the name as well as six consecutive segments after the name. However, for a statistical evaluation, we compared only the 5-s segments immediately before and after the name for both speech envelopes separately, using a Wilcoxon signed-rank test. This statistical evaluation was once done for all name occurrences irrespective of the condition and once for lower and higher name intelligibility condition separately.

## Results

### Detected Names and P3 Component

**FIGURE 3:**
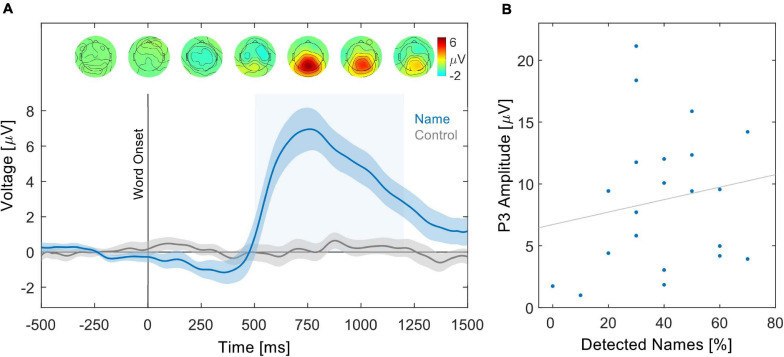
Grand average ERP in response to one’s name. **(A)** Grand average ERP at channel Pz in response to the name (blue) and control word (gray). Shaded areas around the waveforms represent the SE over participants. The positive amplitude deflection in response to the name between 500 and 1200 ms (light blue-shaded rectangle) depicts the P3 component. Above, topographies of the grand average ERP in response to the name are shown at time points ranging from –250 to 1,250 ms relative to name onset. The P3 topography shows a posterior scalp distribution. The ERP in response to the control word did not show a distinct spatial pattern. **(B)** Relation between the percentage of detected names and the individual P3 amplitude in response to the name. Data points represent values of individual participants, whereas the gray line represents the least squares regression.

According to the content questionnaire, participants followed the instruction to attend to their assigned narrative. Participants correctly answered 88.66% (SD 6.82%) of content questions related to their to-be-attended narrative. The low variance across participants in the percentage of correctly answered questions made it inadequate to correlate these behavioral measures with any other obtained measures. All but one participant retrospectively reported that they noticed their name in the to-be-ignored narrative. In line with this, a P3 component in response to one’s name was apparent in the participants’ EEG activity, while no such effect could be observed in response to the control words (Figure 3A). The mean amplitude in the time window from 500 to 1,200 ms was significantly larger in response to the name compared with the control words (*Z* = 3.79, *p* < 0.001). The grand average P3 component in response to the name had a posterior scalp distribution and a latency of 760 ms after name onset. There was a positive, but non-significant, trend between the retrospectively reported number of detected names and the individual P3 amplitude (Figure 3B, rho = 0.37, *p* = 0.0516).

### Validation of Cross-Correlation Approach

**FIGURE 4:**
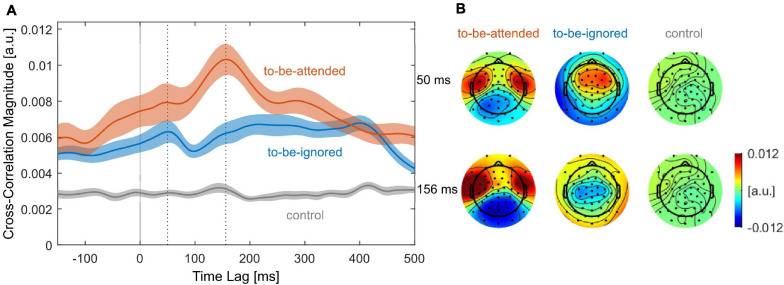
Neural representation of the speech envelopes. **(A)** Grand average cross-correlation magnitude of the to-be-attended (red), to-be-ignored (blue), and control (gray) speech envelopes as a function of time lag. Shaded areas around the grand average functions represent the SE over participants. Dotted vertical lines indicate the time lags of prominent peaks from the to-be-ignored (50 ms) and the to-be-attended (156 ms) speech envelope, respectively. (**B**) Scalp distributions of the grand average cross-correlation values at time lag 50 ms (top) and 156 ms (bottom) separately for each speech envelope.

To validate our cross-correlation procedure, we first performed the cross-correlation with consecutive 5-s segments over the entire duration of the experiment, irrespective of name onsets. The resulting cross-correlation magnitude functions for the respective speech envelopes are shown in Figure 4. The cross-correlation magnitude function of the control speech envelope did not show a clear temporal profile. In agreement with Jaeger et al. (2020), the cross-correlation magnitude function of the to-be-attended speech envelope showed a prominent peak at 156 ms time lag with strongest positive cross-correlation values at bilateral temporal channels. The cross-correlation magnitude function of the to-be-ignored speech envelope showed a prominent peak at 50 ms time lag with strongest positive cross-correlation values at fronto-central channels, hence, corresponding to time lags (Kong et al., 2014; Mirkovic et al., 2019) and to the topography (Jaeger et al., 2020) reported in recent literature. Since the morphology obtained in the grand average cross-correlation magnitude functions was not consistently observed in all individual datasets, we averaged the cross-correlation magnitude values for time lags from 0 to 500 ms to quantify the neural representation of each speech envelope, respectively. Results for this summary score showed a stronger cross-correlation magnitude for the to-be-attended than for the control speech envelope (*Z* = 4, *p* < 0.001) as well as a stronger cross-correlation magnitude for the to-be-ignored than for the control speech envelope (*Z* = 3.96, *p* < 0.001). In addition, the neural tracking of the to-be-attended speech envelope was stronger than the neural tracking of the to-be-ignored speech envelope (*Z* = 1.88, *p* = 0.03).

### Cross-Correlation Magnitude Relative to Name Onset

**FIGURE 5:**
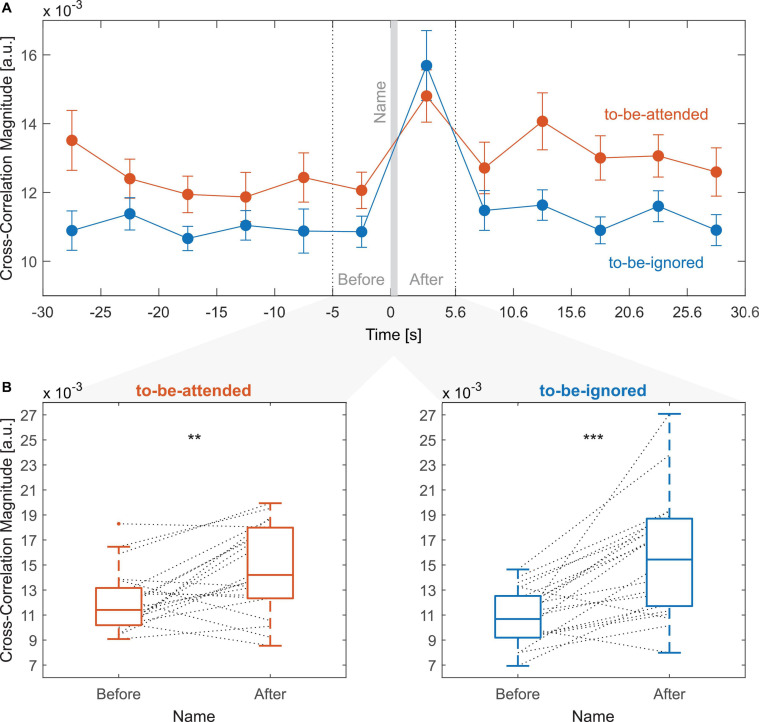
Cross-correlation magnitude values relative to name onset. **(A)** Each data point represents the grand average cross-correlation magnitude value of a 5-s segment relative to the name onset. The individual cross-correlation magnitude values have been calculated as the average of cross-correlation magnitude values over time lags from 0 to 500 ms. Error bars indicate the SE over participants. The 5-s segment from –5 to 0 s (name onset) indicates the time period immediately before the name, whereas the 5-s segment from 0.6 to 5.6 s indicates the time period immediately after the name as marked by the dashed vertical lines. The vertical, gray-shaded area from 0 to 0.6 s indicates the name occurrence. **(B)** Individual cross-correlation magnitude values of the to-be-attended (left) and to-be-ignored (right) speech envelope for time periods immediately before and after the name occurence. Horizontal line within the boxplot indicates the median cross-correlation magnitude value, the edges of the box indicate the 25th and 75th percentile, and the whiskers indicate the extreme values not accounting for outliers. The dashed lines between boxplots connect two data points of a single participant (*p < 0.05, **p < 0.01, and ***p < 0.001).

Figure 5 illustrates that presenting one’s name in the to-be-ignored stream had an influence on the speech envelope tracking of the to-be-attended and to-be-ignored narratives. As expected, the cross-correlation magnitude of the to-be-ignored speech envelope significantly increased from before to after the name occurrence (Figure 5B, *Z* = 3.67, *p* < 0.001). However, in contrast to our hypothesis, the cross-correlation magnitude of the to-be-attended speech envelope did not decrease but increased significantly (Figure 5B, *Z* = 2.69, *p* = 0.007). The increase in cross-correlation magnitude from before to after the name did not differ significantly between the to-be-ignored and to-be-attended speech envelope (Wilcoxon signed-rank test, *Z* = 1.58, *p* = 0.114). Although an increase in the neural tracking of the to-be-attended as well as the to-be-ignored speech stream was apparent for most participants, for some this increase was stronger than for others and still others showed no increase or even a decrease after the name occurrence (Figure 5B).

**FIGURE 6:**
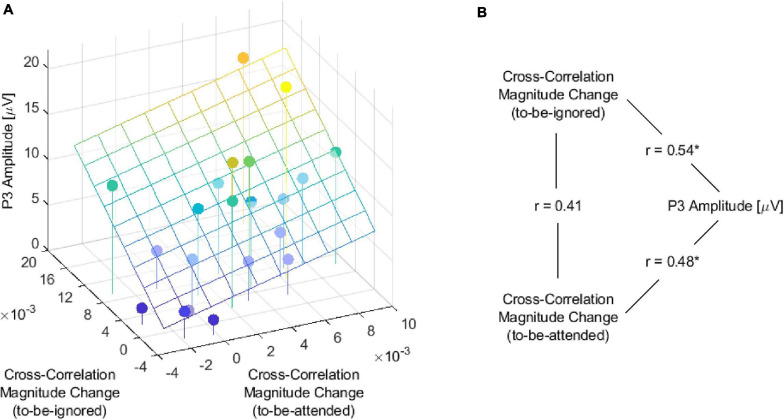
Relation between the P3 amplitude and the cross-correlation magnitude change from before to after the name. **(A)** Cross-correlation magnitude change was determined as the difference between the cross-correlation magnitude values of the 5-s segment before and after the name, once for the to-be-attended and once for the to-be-ignored speech envelope. A positive change indicates an increase in the cross-correlation magnitude from before to after the name. The plane represents the least squares plane. **(B)** Values represent Pearson correlation between the two variables connected by the line (*p < 0.05).

To explore this difference between participants, we investigated the relation between the individual change in cross-correlation magnitude from before to after the name for both the to-be-attended and the to-be-ignored speech envelope and the individual P3 amplitude (Figure 6). Pearson correlations revealed a statistically significant positive relation between the individual P3 amplitude and the individual change in cross-correlation magnitude of the to-be-attended speech envelope (Figure 6B, *r* = 0.48, *p* = 0.03). A numerically stronger positive relation was observed between the individual P3 amplitude and the individual change in cross-correlation magnitude of the to-be-ignored speech envelope (Figure 6B, *r* = 0.54, *p* = 0.01). In addition, the Pearson correlation coefficient between the individual change in cross-correlation magnitude of the to-be-attended and the to-be-ignored speech envelope indicated a positive relation (Figure 6B, *r* = 0.41, *p* = 0.06) which, however, was not significant. Thus, participants with a larger P3 amplitude showed a higher increase in the neural tracking of the to-be-attended and a higher increase in the neural tracking of the to-be-ignored speech envelope after one’s name occurred.

### Speech Intelligibility of the To-Be-Ignored Narrative

**FIGURE 7:**
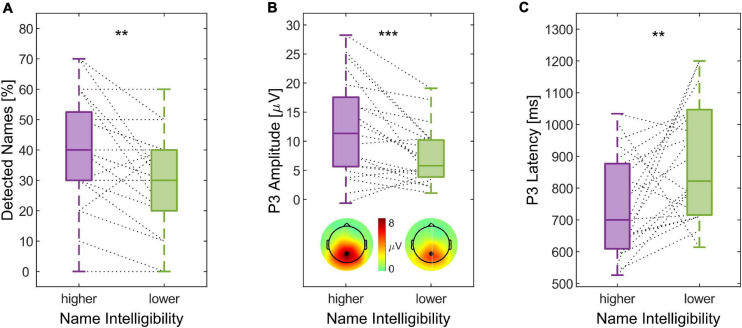
Influence of the to-be-ignored stream’s speech intelligibility on the attention-capturing effect of one’s name. **(A)** Percentage of the subjectively reported number of detected names, **(B)** P3 amplitude including the grand average P3 topography and the channel used for amplitude quantification as inset, and **(C)** P3 latency are shown as boxplots depending on the speech intelligibility of the to-be-ignored speech stream. Dashed lines connect the two condition-dependent measures of the same participant (*p < 0.05, **p < 0.01, and ***p < 0.001).

In line with our hypothesis, reducing the speech intelligibility of the to-be-ignored narrative resulted in a lower number of detected names reported. When name intelligibility was higher, the median number of detected names was 40%, while it was 30% when name intelligibility was lower (Figure 7A). This difference was statistically significant (*Z* = 2.58, *p* = 0.005). Similarly, the P3 amplitude was larger in the higher name intelligibility condition than in the lower name intelligibility condition (Figure 7B, *Z* = 3.27, *p* < 0.001). The P3 latency was significantly shorter when the name intelligibility was higher (Figure 7C, *Z* = −2.62, *p* = 0.004).

When comparing the change in cross-correlation magnitude from before to after the name for the lower and higher name intelligibility condition separately, no difference was found between the conditions (Supplementary Figure 2). In both conditions, there was an increase in the tracking of the to-be-attended narrative as well as an increase in the tracking of the to-be-ignored narrative after one’s name occurred in the to-be-ignored narrative.

## Discussion

We employed speech envelope tracking to investigate the neural correlates of the attentional processes which are evoked when relevant information is presented in an ignored speech stream. As a relevant event, we chose the participant’s name. The presence of a P3 component in response to one’s name provided neurophysiological evidence that participants noticed their name in the to-be-ignored speech stream. The concomitant attentional processes were further investigated with speech envelope tracking. In line with our hypothesis, we observed an increase in the neural tracking of the to-be-ignored speech stream after the name occurrence. However, in contrast to our prediction, the neural tracking of the to-be-attended speech stream increased. In addition, we manipulated the intelligibility of the to-be-ignored speech stream. In the lower name intelligibility condition, less names were detected and a lower P3 amplitude was observed, whereas the neural tracking analysis did not capture any differences between the conditions.

### P3 Amplitude

It is well known that one’s name can capture one’s attention even when uttered by a speaker to which one did not attend to (Moray, 1959; Wood and Cowan, 1995). This has been taken as evidence that ignored speech can be processed up to a semantic level (Bronkhorst, 2015). In the current study, we took great care to blend in the name as good as possible into the to-be-ignored speech stream, so that the name would only stand out due to its semantic content. Yet, as the name utterance was produced by a different speaker, the subtle change in voice characteristics may have contributed to its detection. However, as participants did not report that any other changes in the voice characteristics of the to-be-ignored speaker attracted their attention, we attribute the P3 results to the semantic processing of ignored speech. To ultimately test this, one would have to either use the same speaker for the name as for the to-be-ignored speech stream or add a name other than one’s own as a control.

When the name occurs multiple times in a to-be-ignored speech stream, it remains a challenge to behaviorally assess how many of the name occurrences a participant noticed. To resolve this issue, Moray (1959) proposed the method of shadowing, where participants had to articulate the message from the to-be-attended speech stream while listening to it. Whenever participants were distracted by hearing their name in the to-be-ignored speech stream, they were unable to shadow the message of the to-be-attended speech stream, which was taken as evidence that participants detected their name. However, this approach was rather unsuitable for the current study as we employed neurophysiological measures which in turn would have been contaminated by muscle artifacts when participants spoke (Goregliad Fjaellingsdal et al., 2020). Thus, the behavioral assessment was limited and we could only ask participants after the experiment to retrospectively estimate the number of detected names. Therefore, in addition to this coarse behavioral measure, we used the P3 component as a neurophysiological measure of detected names. Typically, the presence of a P3 component reflects the detection of a relevant event (Polich, 2007). Accordingly, it has consistently been observed that one’s name elicits a P3 component due to its innate relevance (Berlad and Pratt, 1995; Perrin et al., 2005; Holeckova et al., 2006; Eichenlaub et al., 2012). In the current study, the presence of a P3 component provides neurophysiological evidence that participants detected their name in the to-be-ignored speech stream. In addition, the P3 amplitude of each participant’s ERP averaged over all name occurrences was proposed as an estimate of the number of detected names. The rationale behind this was that name occurrences which were not detected did not elicit a P3 component and thus decreased the P3 amplitude in the participants average ERP. This assumption is supported by the positive trend we observed between the retrospectively reported number of detected names and the participant’s P3 amplitude. Further evidence is provided by our finding that a lower number of detected names in the lower name intelligibility condition coincided with a smaller P3 amplitude. Thus, our results demonstrate that the P3 amplitude of the participant’s ERP can be used to make a judgment on the number of detected names. However, it is important to note that the P3 amplitude is influenced by many different factors (Polich, 2007) and that the low sample size limits the correlation analysis between participants.

### P3 Latency

The P3 latency has been shown to reflect the time it takes to detect and evaluate the relevant event (Polich, 2007). In previous studies where participants were only presented with a single auditory stream containing the participant’s name, the P3 latency in response to the name was roughly at 400 ms (Berlad and Pratt, 1995; Perrin et al., 2005). However, in other studies where participants were instructed to attend to a silent movie while being presented with an auditory stream containing the participant’s name, a posterior positivity in response to the name was apparent, which had a latency of 550 (Eichenlaub et al., 2012) and 650 ms (Holeckova et al., 2006), respectively. This posterior positivity was termed parietal positivity, although it has been stated that both terms P3 and parietal positivity may reflect the same component (Eichenlaub et al., 2012). The latency differences between the different experimental paradigms indicate that it takes more time to detect one’s name in an auditory stream a listener is not attending to. This interpretation may explain the rather late P3 latency of 760 ms observed in the current study. Given that the P3 latency of the current study was even later than the ones reported by Eichenlaub et al. (2012) and Holeckova et al. (2006), one is tempted to draw the conclusion that it takes even longer to detect one’s name in a to-be-ignored auditory stream when the to-be-attended stream is also within the auditory modality. This phenomenon could result from the masking properties of sound (Brungart, 2001), especially taking into account that we observed a later P3 latency when the to-be-ignored speech stream was attenuated. However, because other factors and differences in experimental setups can contribute to ERP latency, a direct investigation would be required.

### Neural Tracking of the To-Be-Ignored Speech Stream

When comparing the neural tracking of the to-be-ignored speech stream before and after the participant’s name, an increase was apparent. This confirms that the name had attracted the participant’s attention toward the to-be-ignored speech stream, leading to its transiently enhanced processing. These results complement findings of a recently published study conducted by Huang and Elhilali (2020) where participants were instructed to attend to a tone sequence while a naturalistic auditory background scene was presented, which included physically salient events. In response to highly salient events, an increase in the tracking of the to-be-ignored background was observed. In contrast to these findings, Hambrook and Tata (2019) did not observe such an increase when content from the ignored speech stream intruded on the participants’ perception. However, in their study, the structure and the linguistic content of the to-be-attended and to-be-ignored streams were very similar, which may imply that participants did not even notice that the intruded word came from an ignored stream. In addition, Hambrook and Tata (2019) claimed that the attention-capturing effect of the intruded word might not have lasted long enough to be captured by speech envelope tracking.

### Neural Tracking of the To-Be-Attended Speech Stream

Regarding the neural tracking of the to-be-attended speech, we predicted a lower tracking after presenting one’s name as we expected the name to draw the attentional resources toward the to-be-ignored stream, hence, away from the to-be-attended stream. In fact, both Hambrook and Tata (2019) and Huang and Elhilali (2020) observed such a decrease in the tracking of the to-be-attended stream when stimuli in the to-be-ignored stream caught the participants’ attention. Instead in the current study, a significant increase in the tracking of the to-be-attended speech stream was apparent. A possible explanation may be that hearing one’s name could have acted as a wake-up call which alerted the participants to focus more strongly on their actual task, namely, to attend to their assigned narrative. In fact, such an alerting effect of hearing one’s name has previously been shown to reduce attentional lapses in a monotonous task (Kaida and Abe, 2018). Nevertheless, it is likely that the detection of one’s name in the to-be-ignored speech stream still caused a transient collapse in the comprehension of the to-be-attended speech stream, as previously shown behaviorally (Moray, 1959; Wood and Cowan, 1995). However, we did not observe such an effect in the neural tracking of the to-be-attended speech stream, possibly because the attention-capturing effect of one’s name may have lasted much shorter than the minimum time window of 5 s required for reliable speech envelope tracking using cross-correlation (Jaeger et al., 2020). In fact, according to Conway et al. (2001), it takes less than the duration of two words to reorient back to the to-be-attended stream after one’s name had attracted one’s attention toward the to-be-ignored stream. Thus, with a time resolution of 5 s, a potential transient decrease may have been overcompensated by a subsequent increase in the neural tracking of the to-be-attended speech stream, caused by the alerting properties of one’s name. To ultimately examine the exact temporal dynamics of these attentional processes, a higher temporal resolution for speech envelope tracking would be required. However, one needs to consider that a higher temporal resolution results in less reliable speech envelope tracking due to the low signal-to-noise ratio of EEG (Geirnaert et al., 2020).

### Speech Intelligibility of the To-Be-Ignored Speech Stream

We did not observe a significant effect of attenuating the to-be-ignored stream on the cross-correlation magnitude change after one’s name. For both the lower and higher name intelligibility conditions, the neural tracking of the to-be-attended as well as the neural tracking of the to-be-ignored speech stream increased after presenting one’s name in the to-be-ignored speech stream. The magnitude of this change was not significantly different between the lower and higher name intelligibility conditions, which was unexpected. In the condition-independent analysis, positive correlations between the individual cross-correlation magnitude change of both speech streams and the individual P3 amplitude were observed. Although the reliability of these correlation analyses would have benefited from a larger sample size, we assume that this relation is likely driven by the number of detected names. Thus, we expected the lower number of detected names in the lower name intelligibility condition to coincide with a smaller increase in the neural tracking of both speech streams. However, we did not observe this effect, which may be due to the rather low number of 20 name occurrences per condition, from which only a portion was detected. Unfortunately, increasing the number of name occurrences would not have solved this problem, as it would have reduced the attention-capturing effect of one’s name (Tateuchi et al., 2012).

### Application

In terms of application, the neurophysiological measures presented in the current study could be used to determine the level of attenuating the background of an auditory scene using hearing aids. It is well known that hearing-impaired listeners have major difficulties selectively attending to one speaker in the presence of a complex auditory background (Shinn-Cunningham and Best, 2008). Beamforming algorithms implemented in hearing aids partly provide a solution by attenuating the auditory background to enhance the intelligibility of the target speaker (Doclo et al., 2015). However, in this respect, a fundamental question remains: to what extent can the auditory background be attenuated while preserving the listener’s ability to notice relevant information presented therein? The results of the current study suggest that speech envelope tracking presents a good opportunity to answer this question.

## Conclusion

In conclusion, by using the P3 response to one’s name embedded in a to-be-ignored speech stream, we provide neurophysiological evidence that involuntary attention capture can be observed in competing-speaker paradigms. Furthermore, the speech envelope tracking method of the current study provides evidence for the enhanced transient processing of the to-be-ignored speech stream when relevant information is detected therein. Interestingly, hearing one’s name in the to-be-ignored speech stream does not necessarily seem to distract one from attending to the designated speaker but may function as a wake-up call, resulting in enhanced processing of the to-be-attended speech stream. This phenomenon could be applied to scenarios in which it is essential that participants sustain attention over longer periods of time. We conclude that speech envelope tracking is suitable to assess the transient allocation of attentional resources to highly salient or personally relevant events, presented in an ignored stream. In future studies, this possibility could help unravel the complex dynamics of attentional processes involved in comprehending speech in complex, multi-stream scenarios, which is a daily challenge for many of us.

## Data Availability Statement

The original contributions presented in the study are publicly available. These data can be found here: https://openneuro.org/datasets/ds003516/versions/1.1.0. MATLAB code used to compute the results presented in the current study can be found on GitHub (https://doi.org/10.5281/zenodo.4541039).

## Ethics Statement

The studies involving human participants were reviewed and approved by the Kommission für Forschungsfolgenabschätzung und Ethik, University of Oldenburg, Oldenburg, Germany. The patients/participants provided their written informed consent to participate in this study.

## Author Contributions

BH performed the data acquisition, analyzed the data, and wrote the manuscript to which MJ, SD, KA, and BM contributed with critical revisions. KA implemented the audio presentation within the paradigm. All authors designed the experiment, approved the final version, and agreed to be accountable for this work.

## Conflict of Interest

The authors declare that the research was conducted in the absence of any commercial or financial relationships that could be construed as a potential conflict of interest.

## Footnotes

1 https://doi.org/10.5281/zenodo.4541039
