# Supplementary material for: Are They Calling My Name? Attention Capture Is Reflected in the Neural Tracking of Attended and Ignored Speech
Source: Front Neurosci. 2021 Mar 22;15:643705. doi: 10.3389/fnins.2021.643705 (PMC8019946; doi:10.3389/fnins.2021.643705)
Supplement: Supplementary file 1 [file Data_Sheet_1.PDF]

Supplementary Material

1.1 Supplementary Figures

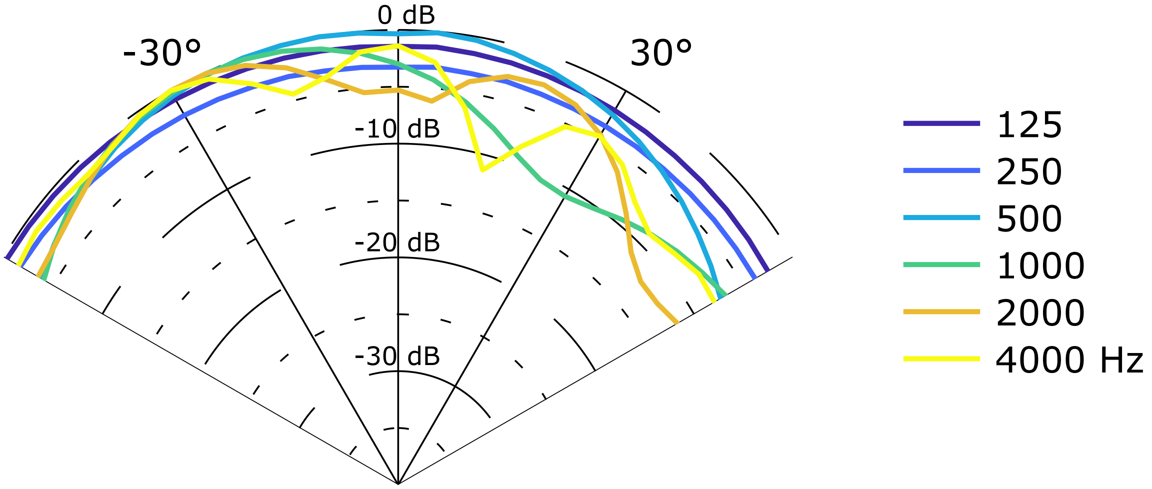

Supplementary Figure S1. Frequency-dependent attenuation of beamforming algorithm. The figure illustrates the attenuation effect of the beamforming algorithm which is directed to the to-be-attended speaker positioned at an angle of -30° in azimuth. Consequently, the to-be-ignored speaker at an angle of +30° in azimuth is attenuated. Colored lines reflect the frequency-dependent level of attenuation for frequencies from 125 to 4000 Hz. As the left and right speaker had a different frequency composition, both speakers were attenuated differently by the same beamformer. According to the root mean square (RMS), when the beamformer was directed to the left speaker located at -30°, the signal-to-noise ratio (SNR) between the to-be-attended (left) and the to-be-ignored (right) speaker was 7.9 dB. When the beamformer was directed to the right speaker located at 30°, the SNR between the to-be-attended (right) and the to-be-ignored (left) speaker was 5.8 dB. Despite this difference in SNR values, the level of attenuation was subjectively perceived as equal, independent of whether the left or right speaker was attenuated.

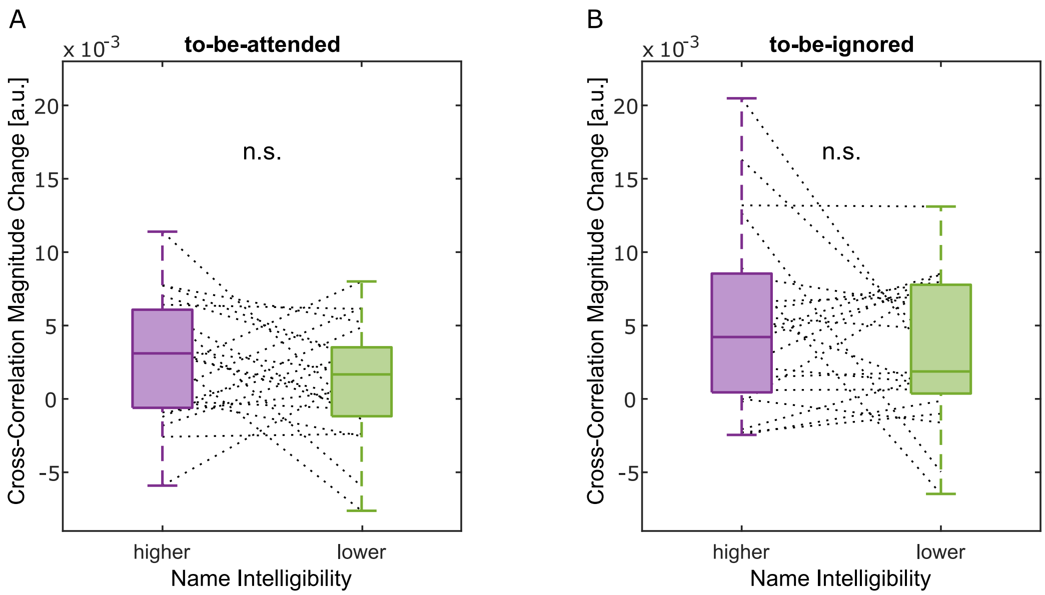

Supplementary Figure S2. Influence of SNR on cross-correlation magnitude changes in response to the name occurrence. Each data point represents the cross-correlation magnitude change from before to after the name occurrence. A positive value reflects an increase in the neural tracking of the respective speech envelope, whereas a negative value reflects a decrease. Dotted lines connect two measures of the same participant. (A) Cross-correlation magnitude change of the to-be-attended speech stream separately for the higher and lower name intelligibility condition (Wilcoxon signed rank test, $Z = 0.92$, $p = 0.36$). (B) Cross-correlation magnitude change of the to-be-ignored speech stream separately for the higher and lower name intelligibility condition (Wilcoxon signed rank test, $Z = 0.3$, $p = 0.77$).
